# The Mesh of Civilizations in the Global Network of Digital Communication

**DOI:** 10.1371/journal.pone.0122543

**Published:** 2015-05-29

**Authors:** Bogdan State, Patrick Park, Ingmar Weber, Michael Macy

**Affiliations:** 1 Stanford University, Stanford, California, United States of America; 2 Cornell University, Ithaca, New York, United States of America; 3 Qatar Computing Research Institute, Doha, Qatar; Instituto de Fisica Interdisciplinar y Sistemas Complejos IFISC (CSIC-UIB), SPAIN

## Abstract

Conflicts fueled by popular religious mobilization have rekindled the controversy surrounding Samuel Huntington’s theory of changing international alignments in the Post-Cold War era. In *The Clash of Civilizations*, Huntington challenged Fukuyama’s “end of history” thesis that liberal democracy had emerged victorious out of Post-war ideological and economic rivalries. Based on a top-down analysis of the alignments of nation states, Huntington famously concluded that the axes of international geo-political conflicts had reverted to the ancient cultural divisions that had characterized most of human history. Until recently, however, the debate has had to rely more on polemics than empirical evidence. Moreover, Huntington made this prediction in 1993, before social media connected the world’s population. Do digital communications attenuate or echo the cultural, religious, and ethnic “fault lines” posited by Huntington prior to the global diffusion of social media? We revisit Huntington's thesis using hundreds of millions of anonymized email and Twitter communications among tens of millions of worldwide users to map the global alignment of interpersonal relations. Contrary to the supposedly borderless world of cyberspace, a bottom-up analysis confirms the persistence of the eight culturally differentiated civilizations posited by Huntington, with the divisions corresponding to differences in language, religion, economic development, and spatial distance.

Have social media created a global village that spans cultural differences and traditional borders? For most of the postwar period, research on international alignments was informed by World Systems Theory, an approach that emphasized the influence of North-South economic divisions and East-West ideological divisions **[1]**. Following the collapse of the Soviet Union, Fukuyama **[2]** proposed that those divisions had culminated in the triumph of liberal democratic systems, signaling the “end of history.” In *The Clash of Civilizations* **[3]**, Fukuyama’s mentor, Samuel Huntington, acknowledged that “the fundamental source of conflict in this new world will not be primarily ideological or primarily economic,” but he challenged Fukyama’s prediction of global consensus:

The great divisions among humankind and the dominating source of conflict will be cultural. Nation states will remain the most powerful actors in world affairs, but the principal conflicts of global politics will occur between nations and groups of different civilizations. The clash of civilizations will dominate global politics. The fault lines between civilizations will be the battle lines of the future.

The world has undergone substantial changes since Huntington made this prediction in 1993, including rapid economic growth in South Asia and South America and global diffusion of social media connecting the world’s population. Moreover, the evidence for Huntington's theory of international global alignment was largely top-down, based on the political and economic relations among culturally differentiated nation states. The growing availability of digital traces of human communications now makes it possible to revisit Huntington's thesis by taking a bottom-up view, based on interpersonal interactions among millions of individuals instead of the alignments of state actors. Have these online interactions created a global village that spans not only the old Cold War divisions but Huntington’s cultural fault lines as well?

To find out, we used hundreds of millions of anonymized email and Twitter communications among tens of millions of worldwide users to map global patterns of transnational interpersonal communication. We measure the density, not the content, of interpersonal communication, and our findings do not address Huntington’s highly controversial warning of the potential for conflict between cultures. Nevertheless, the observed patterns provide a unique angle on changing global alignments in the Post-Cold War era.

Our work builds on a study by Leskovec and Horvitz of the global flows of online Instant Messaging (IM) **[4],** extending that research in four important ways. First, we measure the density of social ties, not the density of message traffic. The “fault lines of civilizations” are indicated not by the number of messages two people exchange across borders but by the number of individuals in each country who exchange messages with one another. Second, we measure the density of social ties after taking into account differences in language, geo-location, Cold War alignment, economic development, and religion. (NB: In addition to analyzing communication densities between country pairs, Leskovec and Horvitz also measured global properties of the social network among pairs of individuals, but they did not use the latter to analyze social densities between country pairs. They also found that between-country communication densities were highly correlated with language and spatial distance, but they did not measure communication densities relative to the expected density given similarities of language and geo-location.) Third, we use multiple platforms (Yahoo! email and Twitter) to provide greater robustness and to avoid the possibility that our results are artifacts of the idiosyncrasies of a particular service. Twitter differs from email in two ways that are relevant to our study: most email exchanges are dyadic while Twitter messages are publicly visible, and the Twitter platform makes it much easier for users to discover one another, thereby reducing the tendency for online interactions to reflect pre-existing relationships. Fourth, we correct for internet access and market penetration, without which online traffic flows confound the strength of interpersonal ties with the popularity of a given communication medium. By taking into account differences in Internet access and market penetration, we are able to estimate the global network of social ties derived from the interpersonal flows of Internet communications.

We measured communication density based on the number of observed bi-directed ties between users in two countries, relative to the maximum possible number of such ties, given the number of users in each country and the number of people with Internet access. (NB: The email analysis was based on anonymized geo-tagged edge lists. Prior to the analysis, the edge lists were anonymized as follows: Individual email accounts were assigned a random numeric identifier, after which the original email addresses were deleted. The email message content was not accessed or used for this study. Other than country, the edge lists we analyzed contained no individually identifiable information. The project was approved by the Cornell University IRB. The Twitter dataset was provided to us already anonymized by the team that collected it [5]. Scripts for the processing of that dataset are available from http://dx.doi.org/10.6084/m9.figshare.1304572.)

A bi-directed email tie exists between two users if at least two messages are exchanged, one in each direction. A bi-directed Twitter tie exists if two users each follow the other. We defined pairwise communication density as the ratio between the observed number of bi-directed ties and the number of expected ties, given the number of subscribers and the number of Internet users.

We obtained qualitatively similar results for email and Twitter communication densities and therefore report only the combined density measures for the two platforms. We also measured densities net of geographic proximity, shared language, population size, economic development, and colonial history.

## Results

**Fig 1:**
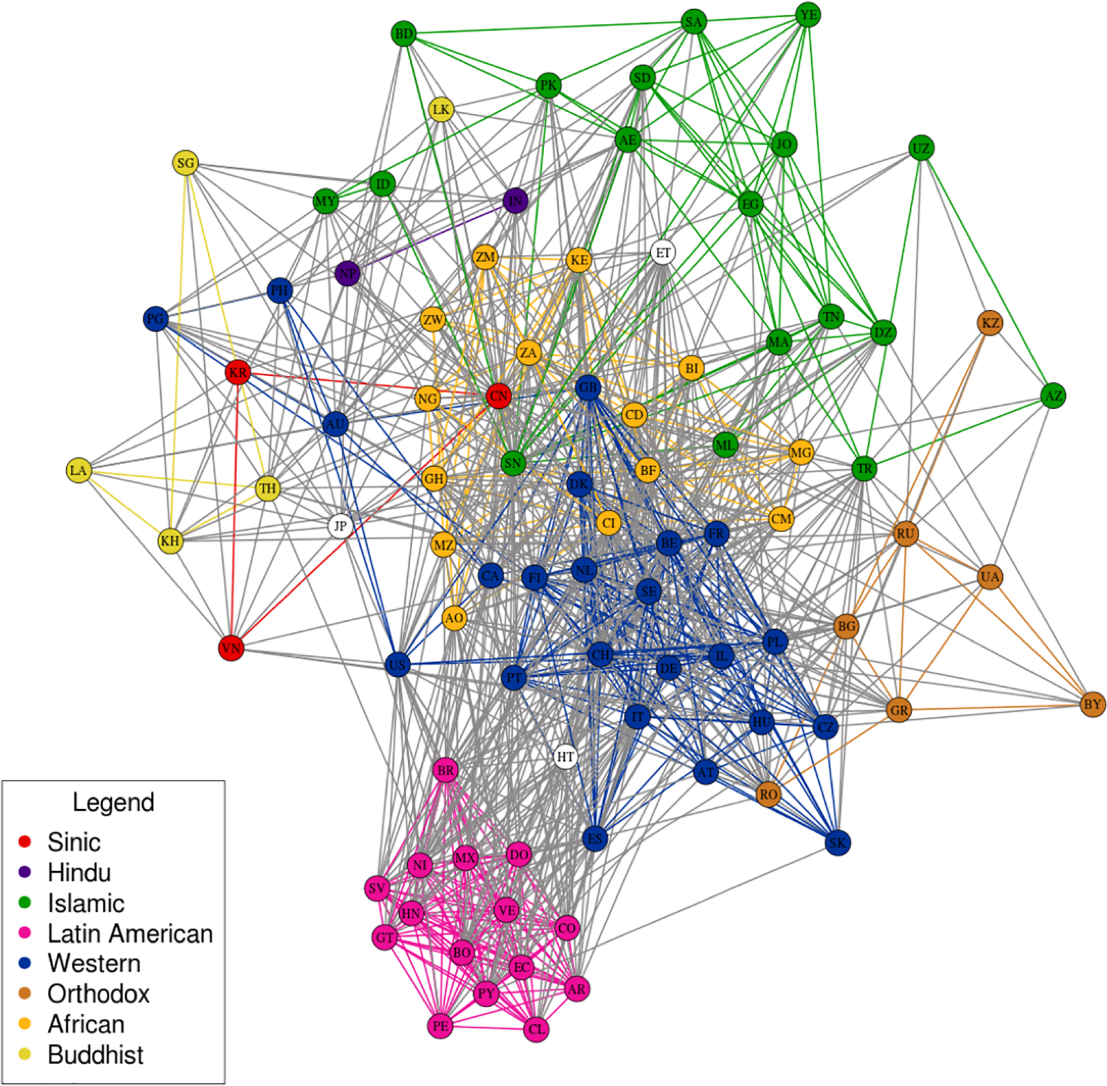
Countries are clustered based on the difference between observed and expected density of social ties in 90 countries with population above 5m, based on interpersonal email and Twitter communication. To facilitate visualization, only the 1000 densest social ties are illustrated. Countries are color-coded according to location in one of Huntington’s eight civilizations. Fig 1 shows that the clusters are highly color specific, with a remarkably close correspondence to Huntington’s eight civilizations. Network layout using the Fructherman-Reingold algorithm [8].

Fig 1 visualizes the communication network among all possible pairs of 90 countries with population above 5M, where the edge weights correspond to the adjusted density measure, derived from the difference between observed and expected values. (NB: A full list of countries included in the analysis is available in S1 Table. Robustness checks revealed qualitatively similar results when countries with at least 1M inhabitants were included. However a 1M threshold is highly problematic, as many pairs of small countries involve too few observations of interpersonal communication for reliable estimates of communication density. See S3 Table for details.) A positive (negative) weight indicates density greater (less) than that expected, given the number of subscribers and the number of Internet users in the country. The visualization locates countries within clusters based solely on network density. (NB: For visualization purposes, we display only the 1000 densest edges in the network, but all edges (regardless of density) were included in the clustering algorithm used to locate countries in the two-dimensional visual array.) Countries are color-coded according to location in one of Huntington’s eight civilizations, as demarcated by Russett, Oneal and Cox **[6, 7]**. The clustering algorithm takes into account only the relative density of ties between users in each country pair, and not the putative civilization (“color”) of a country. Nevertheless, Fig 1 shows that the edge-defined clusters are highly color specific, with very few countries with different colors located in the same cluster. The figure shows a remarkably close correspondence between Huntington’s eight civilizations and the global pattern of interpersonal communication among email and Twitter users worldwide. The results also reveal internal divisions in the Islamic world that Huntington had recognized as well. Finally, the dense Latin American cluster supports Huntington’s contention that this region should be recognized as separate from the West.

Two caveats should be carefully noted. First, while the observed communication densities are consistent with Huntington’s predictions, this does not rule out alternative theories about international alignments whose predictions might also fit the observed pattern. If we begin with Huntington’s theoretically derived groupings and then compare these with the observed densities, the fit is impressive. However, suppose we were to begin empirically and then proceed inductively to look for an explanation. If the nodes in Fig 1 were monochromatic, there are any number of possible faces that we might see in the clouds. We therefore compared Huntington’s assignments to those made by two community detection algorithms for weighted undirected graphs (computed using the igraph R package, **[9]**): the Spinglass algorithm **[10]** and the “fastgreedy” algorithm proposed by Clauset, Newman and Moore **[11]**. These algorithms differ from Huntington’s in that they are entirely empirical rather than based on a theory of international alignments. The two empirical algorithms detect fewer communities than the number proposed by Huntington (three with “fastgreedy” and four with Spinglass), which reflects the very dense nature of the weighted graph. An examination of the Rand index **[12]** reveals that the best agreement occurs between Huntington’s assignments and the Spinglass algorithm, with about two-thirds of all pairs of countries in agreement. The “Fastgreedy” community detection algorithm has slightly less agreement with Huntington’s assignments, with 59% of all pairs in the same community, which is also the extent of agreement between “Fastgreedy” and the Spinglass algorithm. The comparisons reveal roughly as much disagreement between the two community detection algorithms as between Huntington’s assignments and each of the two algorithms. Thus, while Huntington’s are not the only divisions possible, they remain empirically plausible when compared with two patterns derived from empirical optimization.

Second, while Fig 1 provides striking visual confirmation of Huntington’s predicted global alignments, the clustering is not a statistical test of the hypothesized divisions or the underlying causal mechanism that he proposed—cultural similarities and differences. In addition, the reported densities could be nothing more than an artifact of civilization-specific shared language as a requisite for communication. To address these issues, we used an MRQAP model **[13]** of network density as a function of economic, geo-spatial, and cultural differences, using the countries in Fig 1 as the units of analysis. (NB: MRQAP models were estimated using netlm procedure in the sna R package [14]. See Methods Section for details on the construction of the measures and the data imputation algorithms.) MRQAP provides robust covariate estimates for network autocorrelated data **[15].** The response measure is the density of between-country ties, identical to the measure used to construct the network visualization in Fig 1. Results are reported in Table 1. Model 0 reports the zero-order MRQAP estimate for the bivariate relationship between shared civilization and rescaled density. Model 1 shows that the density differences between civilizations persist net of shared language. The decline in the coefficient from 1.77 (*p* <. 001) to 1.28 (*p* <. 001) shows that most of the differences between civilizations cannot be attributed to language.

**Table 1:** MRQAP Decomposition of Pairwise Correspondence Between Civilization and Communication Density.

	Model 0	Model 1	Model 2	Model 3	Model 4
Intercept	-1.58*	-1.67*	-1.68*	5.97*	6.37*
Common Civilization	1.77*	1.28*	1.24*	0.15	0.10
Shared Language		2.10*	2.00*	1.89*	1.71*
Internat’l Alignment					
Western Bloc			0.81*		0.97*
Eastern Bloc			0.61		-0.14
Non-Aligned			-0.42^**+**^		-0.28
Colonial Ties			1.74*		1.55*
Commonwealth Ties			-0.05		0.07
Cultural Alignment					
Religion				-0.17*	-0.15*
Ln Distance (km)				-0.82*	-0.87*
Shared Border				0.63*	0.52*
Model Fit					
Adjusted *R* ^2^	0.11	0.19	0.22	0.33	0.35
F-statistic	502.1	456.0	160.7	381.4	217.5
dF	1, 4003	2, 4002	7, 3977	5, 3999	10, 3994

*p <. 01,

+ *p* <. 05,

*N* = 4004 country pairs.

Model 2 tests Huntington’s argument that the North-South and East-West divisions of an earlier era are no longer the principle axes of global alignments. We used measures of unequal economic development and shared colonial histories to capture the neo-colonial dimension. For cold war alignments, we coded countries based on their pre-1989 membership in mutual defense pacts with each of the two superpowers, membership in the Non-Aligned Movement, and unaffiliated. (The codings are listed in the Supplemental S2 Table). The results show virtually no change in the effect of common civilization when these “World Systems” measures are controlled, which is consistent with Huntington’s theory that the North-South and East-West alignments of the cold war era are no longer relevant.

Model 3 tests Huntington’s alternative explanation, that global alignments increasingly reflect cultural divisions, centered mainly on religion **[16]**. We measured religion using a religious distance measure based on each country’s composition with respect to major world religions (the procedure is detailed in the Methods section). In addition to shared language and religion, we used physical proximity as a proxy for cultural similarity, based on numerous studies showing that cultural features diffuse spatially **[17, 18, 19, 20, 21]**. The results show that these measures account for most of the effect associated with civilization, as the coefficient declines to. 15 and is no longer statistically significant (*p* >. 05). All the cultural measures are significant (*p* <. 01), which leads us to conclude that the global alignments illustrated in Fig 1 correspond primarily with cultural similarities and differences, especially religion.

## Discussion

Huntington’s research on changing international alignments relied on macrosocial measures of relations between nation states. The digital records of global email and Twitter communication offers an unprecedented opportunity to re-analyze these alignments by observing tens of millions of interpersonal interactions worldwide. A bottom-up analysis of the density of online social ties derived from the global flow of interpersonal email and Twitter communication fits closely with the predicted alignment of nation states in Huntington's model. Contrary to the borderless portrayal of cyberspace, online social interactions do not appear to have erased the fault lines Huntington proposed over a decade before the emergence of social media.

There is, however, an important difference with Huntington’s analysis. Critics have questioned whether divisions among Huntington’s civilizations are a predictor of ethnic conflict **[15]**, and this criticism applies as well to email and Twitter communication. There is no reason to assume that conflict is inevitable between countries with lower density of social interaction. For example, research on the impact of social media in the Arab Spring suggests that digital communications may facilitate divisions within civilizations as much or more than divisions between [**22**]. The evidence we present shows the "mesh” of civilizations, not the "clash.”

## Materials and Methods

### Network Density

We use bi-directed communications on Yahoo! email and Twitter to estimate the pairwise density of Internet-mediated social ties between countries. Although our data come from two of the world's largest Internet-mediated communication services, the unadjusted density is biased by the fraction of Internet users who subscribe to each service. For each pair of countries *i* and *j*, we corrected for uneven market penetration by estimating the number of Internet-mediated ties between the two countries (NB: Internet penetration rates were obtained from internetworldstats.com), taking into account the observed number of bidirected communication ties (*T*_*ij*_), the number of Internet users (*U*_*i*_ and *U*_*j*_), and the number of subscribers in each country in our data set (*S*_*i*_ and *S*_*j*_):

$${T\prime }_{ij}=T_{ij}\;\left(\frac{U_{i}}{S_{i}}\frac{U_{j}}{S_{j}}\right)$$ (1)

Eq 1 scales up the observed tie count in country *i* by the product of the inverse proportions of Internet users who are subscribers in *i* and *j*, in order to account for social ties that are missing in our data: between subscribers in *i* and non-subscribers in *j* (e.g., when a Yahoo! subscriber emails a Gmail subscriber), between subscribers in *j* and non-subscribers in *i*, and between non-subscribers in *i* and *j*.

We do not scale up to account for between-country social ties between individuals who do not have Internet access and therefore can only communicate via telephone or regular post. To do so would almost certainly overstate the number of social ties for the simple reason that international telephone rates are costly, while internet-mediated communication is free. We confirmed this indirectly in two ways. First, we replicated our analysis using just email ties and just Twitter ties and found that the observed international alignments were nearly identical, which suggests that we would find similar patterns for other Internet-based services (like Gmail or Facebook) if these data were available. Second, we replicated our analysis based on rescaling that included individuals who do not have Internet access (by replacing *U* in Eq 1 with the population sizes of *i* and *j*) and the results were a random pattern. These two tests suggest that Internet users are the appropriate population from which to estimate the density of social ties using data on interpersonal communication.

The density measure also needs to take into account the expected number of ties (${\hat{T}}_{ij}$) under the assumption of random communication, given the distribution of international ties. We calculated the expected number using an adjustment procedure that is equivalent to randomly “rewiring” the rescaled network (from Eq 1) while holding constant the degree of the nodes. We then derived tie density as the log ratio of the estimated to expected number of communication ties:

$$D_{ij}=\text{log}\frac{{T\prime }_{ij}}{{\hat{T}}_{ij}}$$ (2)

D_ij_ corresponds to the logged relative risk as a measure of the extent to which the observed and rescaled number of ties (*T*′_*rs*_) diverges from our expected count under randomness (${\hat{T}}_{ij}$). We choose the relative risk for its straightforward interpretation that takes a random-connection scenario as the reference point. Because the relative risk ratio varies between 0 and infinity, we take the logarithm to rescale values as positive or negative as the MRQAP model specification demands. A large positive value indicates “attraction” and more ties than expected under the random model, and a large negative value indicates “repulsion” and fewer ties than expected.

We measured *D*_*ij*_ separately for Yahoo! Mail and Twitter and then averaged the two values. For country pairs with too few users for a reliable measure of density on either email or Twitter, we used only the density for the other service. For country pairs with too few users for a reliable measure of density on both email and Twitter, we set the rescaled density for that country pair equal to the minimum observed rescaled density.

### Geolocation

Yahoo! Mail users were geolocated using the same procedure described in **[19]**, whereas Twitter users were assigned country-level location through the same protocol used in **[23]**.

### Religious Divergence

Data on the religious composition of world countries (for Atheism, Buddhism, Christianity, Hinduism and Islam) was obtained from the *World Religions Database* **[24]**. For every pair of countries (*i*, *j*) we defined religious divergence as the Jensen-Shannon divergence of their religious composition:

$$\text{div}\left(i,j\right)=\frac{1}{2}\sum_{r}\;\text{log}\frac{p^{\text{r}}{}_{\text{i}}}{p^{\text{r}}{}_{\text{j}}}\text{log}p^{\text{r}}{}_{\text{i}}+\frac{1}{2}\sum_{r}\;\text{log}\frac{p^{\text{r}}{}_{\text{j}}}{p^{\text{r}}{}_{\text{i}}}\text{log}p^{\text{r}}{}_{\text{j}}$$ (3)

Here *r* indexes the world religion and *i* and *j* index the country pair.

### Robustness Checks

We exclude countries below a minimum population threshold for obtaining reliable estimates of network density. We report in S3 Table the robustness of the coefficient for “common civilization” across a range of threshold levels. The results reveal a similarly steep decline in the coefficient between Models 2 and 3 regardless of threshold. The only qualitative change is that the coefficient remains significant in Models 3 and 4 (P <. 01) when we include countries with at least 1M inhabitants, which we attribute to outliers in the cultural predictors for very small countries.

## Supporting Information

S1 Table Countries included in analysis. (PDF)

S2 Table Political alliance codings. (PDF)

S3 Table Common civilization coefficient for different levels of minimum population threshold. (PDF)
