# Supplementary material for: The Mesh of Civilizations in the Global Network of Digital Communication
Source: PLoS One. 2015 May 29;10(5):e0122543. doi: 10.1371/journal.pone.0122543 (PMC4449232; doi:10.1371/journal.pone.0122543)
Supplement: S1 Table — (PDF) [file pone.0122543.s001.pdf]

The Mesh of Civilizations:
Supplemental Material

Table S1. **Countries included in analysis**

No.	Country	ISO 3166-2 Code
1	United Arab Emirates	AE
2	Angola	AO
3	Argentina	AR
4	Austria	AT
5	Australia	AU
6	Azerbaijan	AZ
7	Bangladesh	BD
8	Belgium	BE
9	Burkina Faso	BF
10	Bulgaria	BG
11	Burundi	BI
12	Bolivia, Plurinational State Of	BO
13	Brazil	BR
14	Belarus	BY
15	Canada	CA
16	Congo, The Democrat. Republic Of	CD
17	Switzerland	CH
18	Cote D'Ivoire	CI
19	Chile	CL
20	Cameroon	CM
21	China	CN
22	Colombia	CO
23	Czech Republic	CZ
24	Germany	DE
25	Denmark	DK
26	Dominican Republic	DO
27	Algeria	DZ
28	Ecuador	EC
29	Egypt	EG
30	Spain	ES
31	Ethiopia	ET
32	Finland	FI
33	France	FR
34	United Kingdom	GB
35	Ghana	GH
36	Greece	GR
37	Guatemala	GT
38	Honduras	HN
39	Haiti	HT
40	Hungary	HU

41	Indonesia	ID
42	Israel	IL
43	India	IN
44	Italy	IT
45	Jordan	JO
46	Japan	JP
47	Kenya	KE
48	Cambodia	KH
49	Korea, Republic Of	KR
50	Kazakhstan	KZ
51	Lao People's Democratic Republic	LA
52	Sri Lanka	LK
53	Morocco	MA
54	Madagascar	MG
55	Mali	ML
56	Mexico	MX
57	Malaysia	MY
58	Mozambique	MZ
59	Nigeria	NG
60	Nicaragua	NI
61	Netherlands	NL
62	Nepal	NP
63	Peru	PE
64	Papua New Guinea	PG
65	Philippines	PH
66	Pakistan	PK
67	Poland	PL
68	Portugal	PT
69	Paraguay	PY
70	Romania	RO
71	Russian Federation	RU
72	Saudi Arabia	SA
73	Sudan	SD
74	Sweden	SE
75	Singapore	SG
76	Slovakia	SK
77	Senegal	SN
78	El Salvador	SV
79	Thailand	TH
80	Tunisia	TN
81	Turkey	TR
82	Ukraine	UA
83	United States	US
84	Uzbekistan	UZ

85	Venezuela, Bolivarian Republic Of	VE
86	Vietnam	VN
87	Yemen	YE
88	South Africa	ZA
89	Zambia	ZM
90	Zimbabwe	ZW
