# Supplementary material for: The Mesh of Civilizations in the Global Network of Digital Communication
Source: PLoS One. 2015 May 29;10(5):e0122543. doi: 10.1371/journal.pone.0122543 (PMC4449232; doi:10.1371/journal.pone.0122543)
Supplement: S2 Table — (PDF) [file pone.0122543.s002.pdf]

Table S2. Political alliance codings

**Eastern Bloc**		
Azerbaijan	Czech Republic	Russia
Belarus	Hungary	Slovakia
Bulgaria	Kazakhstan	Ukraine
Cambodia	Poland	Uzbekistan
China	Romania	Vietnam
**Western Bloc**		
Argentina	Ecuador	Pakistan
Australia	El Salvador	Paraguay
Belgium	France	Philippines
Bolivia	Guatemala	Portugal
Brazil	Haiti	Thailand
Canada	Honduras	Turkey
Chile	Italy	U.K.
Colombia	Japan	U.S.
Denmark	Mexico	Venezuela
Dominican Republic	Nicaragua	
**Non-Aligned Movement**		
Algeria	Ethiopia	Nigeria
Angola	Ghana	Pakistan
Argentina	India	Peru
Bangladesh	Indonesia	Saudi Arabia
Benin	Jordan	Senegal
Bolivia	Kenya	Singapore
Burkina Faso	Laos	Sri Lanka
Cameroon	Madagascar	Sudan
Chile	Malawi	Tunisia
Colombia	Malaysia	U.A.E.
Congo, DRC	Morocco	Yemen
Cote d'Ivoire	Nepal	Zambia
Ecuador	Nicaragua	Zimbabwe
Egypt		
