# Supplementary material for: The Mesh of Civilizations in the Global Network of Digital Communication
Source: PLoS One. 2015 May 29;10(5):e0122543. doi: 10.1371/journal.pone.0122543 (PMC4449232; doi:10.1371/journal.pone.0122543)
Supplement: S3 Table — (PDF) [file pone.0122543.s003.pdf]

Table S3. Common civilization coefficient for different levels of minimum population threshold.

Threshold	Model 0	Model 1	Model 2	Model 3	Model 4
1m	1.83*	1.40*	1.32*	0.36*	0.32*
5m	1.77*	1.28*	1.23*	0.15	0.09
10m	1.54*	1.07*	1.06*	0.05	0.02
20m	1.15*	0.85*	0.87*	0.03	0.03

*Significant at .01 level. See Table 1 in main text for model specification.
